# Follicular Mucinosis in a Young Male: A Case Report and Literature Review

**DOI:** 10.7759/cureus.97338

**Published:** 2025-11-20

**Authors:** Nourah Alazemi, Dana B Sakhi, Fatema Shamsaldeen, Humoud Al-Sabah

**Affiliations:** 1 General Practice, Kuwait Institute for Medical Specializations (KIMS), Ministry of Health, Kuwait City, KWT; 2 Family Medicine, Al Salam Al Assima Hospital, Kuwait City, KWT; 3 Internal Medicine, Farwaniya Hospital, Kuwait City, KWT; 4 Dermatopathology, As'ad K. Al-Hamad Dermatological Center, Kuwait City, KWT

**Keywords:** alopecia mucinosa, cutaneous t-cell lymphoma, follicular mucinosis, mucin deposition, mycosis fungoides

## Abstract

Follicular mucinosis (FM), also known as alopecia mucinosa, is an uncommon cutaneous mucinosis defined by the deposition of glycosaminoglycan-rich mucin within the pilosebaceous unit. This abnormal accumulation primarily affects the outer root sheath of hair follicles and sebaceous glands, resulting in follicular epithelial degeneration, inflammatory infiltration, and disruption of follicular architecture. Clinically, FM manifests as erythematous papules, indurated plaques, and localized areas of alopecia. We present the case of a 21-year-old male with a five-month history of a localized forehead lesion that demonstrated characteristic clinical and histopathologic features of FM, followed by a concise review of relevant literature.

## Introduction

Follicular mucinosis (FM), also known as alopecia mucinosa, is a rare inflammatory dermatosis characterized by mucin deposition within the outer root sheath and sebaceous glands, leading to follicular degeneration and alopecia. This condition may present either as a primary idiopathic disease or as a secondary process associated with benign inflammatory dermatoses and hematologic malignancies, particularly mycosis fungoides (MF) [1]. 

Clinically, FM manifests as grouped follicular papules, indurated plaques, and non-scarring alopecia, often accompanied by erythema and scaling. While spontaneous remission may occur within months to years in primary FM, secondary FM has a more protracted course, and its association with lymphoma carries a poorer prognosis [2].

Histopathology remains the cornerstone of diagnosis, revealing folliculotropic lymphocytic infiltrates, reticular epithelial degeneration, and mucin accumulation within hair follicles. Alcian blue staining is often required to highlight mucin deposits. Due to its variable course, unpredictable prognosis, and potential for malignant transformation, FM poses significant diagnostic and management challenges [3].

## Case presentation

**Figure 1:**
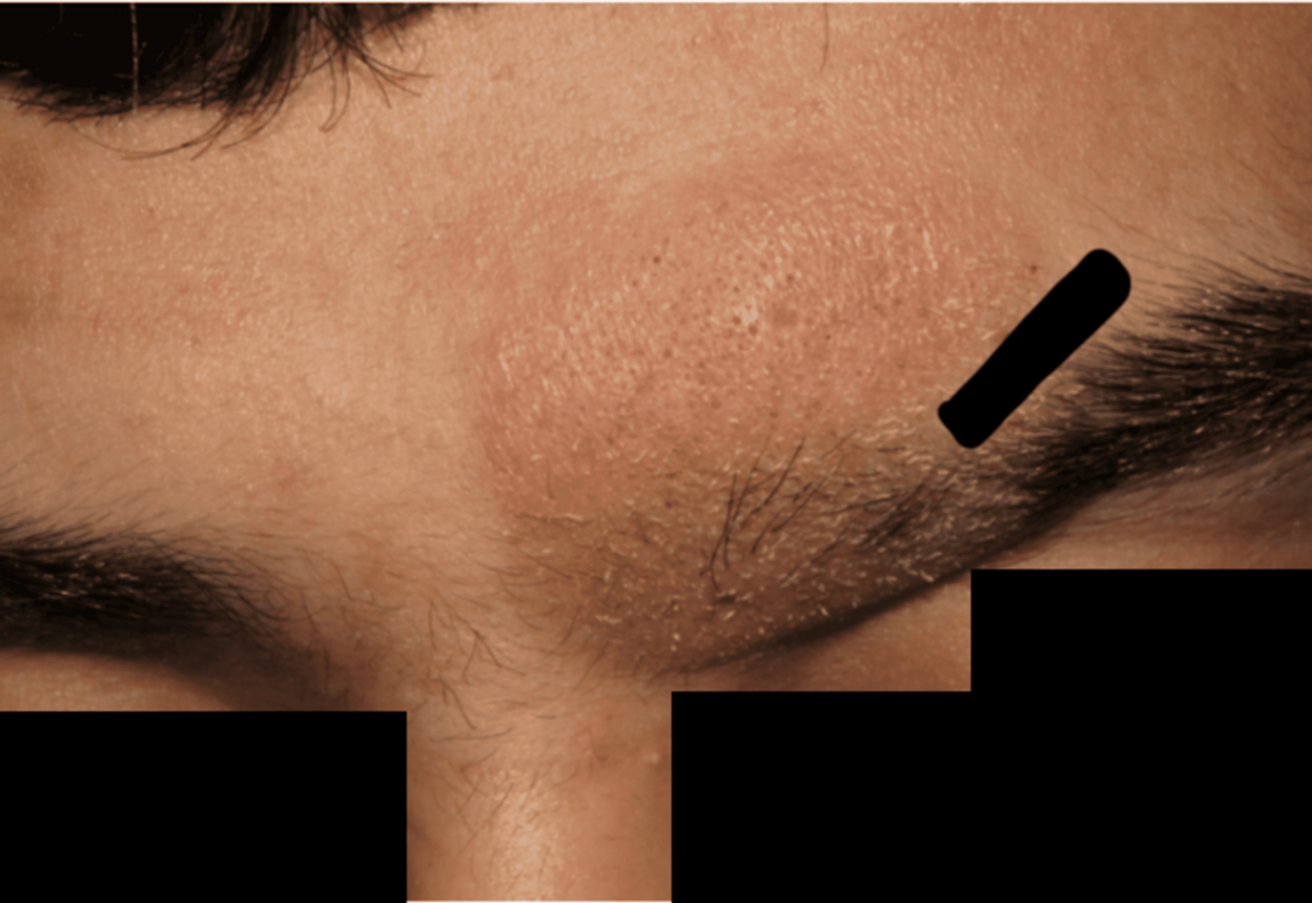
Clinical photograph showing an erythematous, alopecic plaque on the forehead of the patient with eyebrow involvement.

A 21-year-old previously healthy male presented to the Dermatology Clinic at Al Jahra Hospital, Al Jahra, Kuwait, with a five-month history of a skin lesion on the left side of his forehead. Over time, the lesion had gradually expanded to involve part of the left eyebrow, accompanied by partial localized hair loss (Figure 1). The patient reported no pain, pruritus, discharge, or preceding trauma, and denied the use of any topical products or medications on the affected area. There was no history of similar lesions elsewhere, and no constitutional symptoms such as fever, night sweats, or weight loss.

Physical examination revealed a well-defined, erythematous, infiltrated plaque measuring approximately 2 × 3 cm over the left forehead and lateral eyebrow region, with marked loss of eyebrow hair within the involved area (Figure 1). The surrounding skin appeared normal, with no evidence of scaling, ulceration, or secondary infection. No other cutaneous or mucosal lesions were noted. Regional lymph nodes were not palpable.

**Figure 2:**
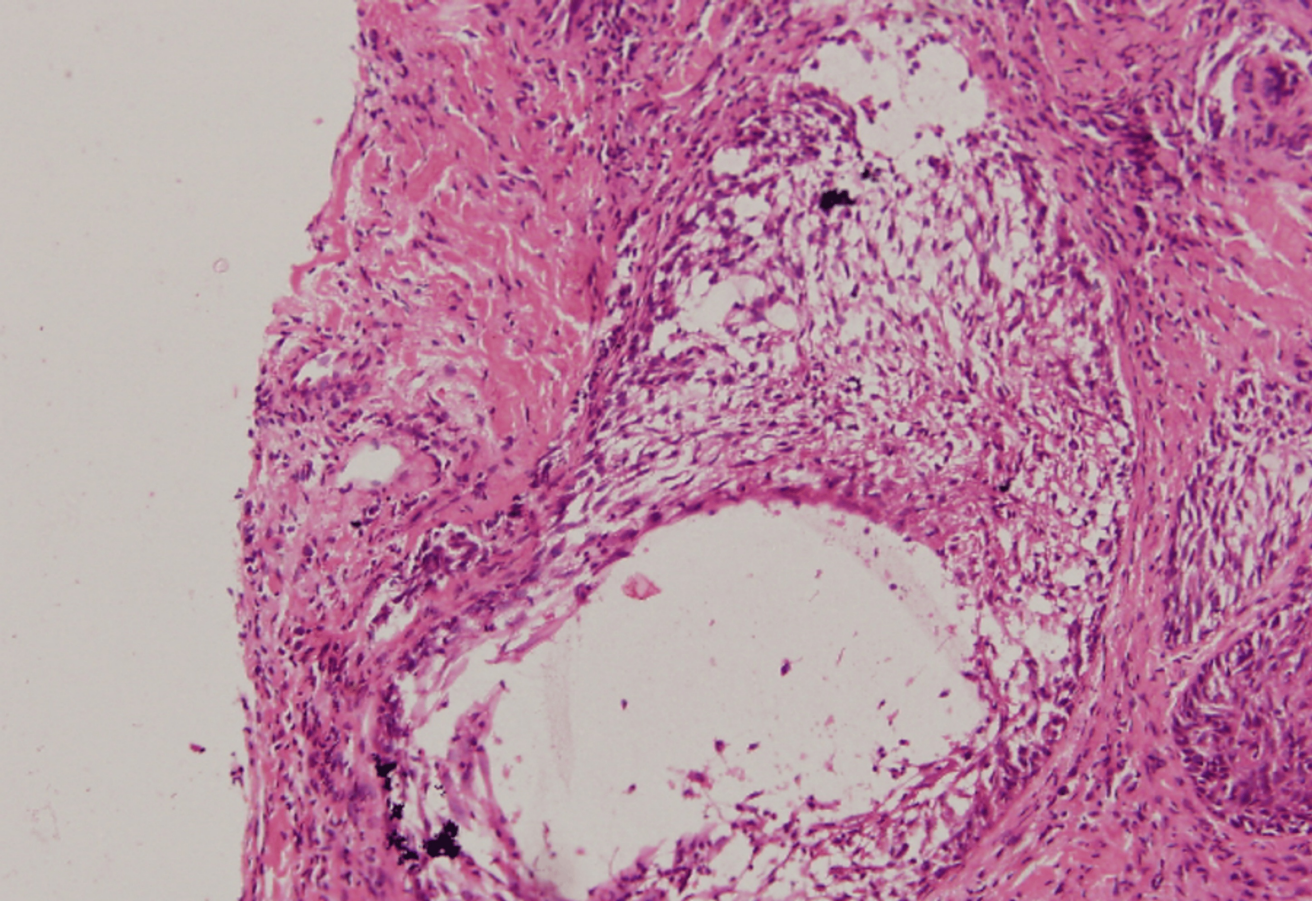
Histopathology (H&E stain) demonstrating a lymphocytic infiltrate with intra- and perifollicular involvement, along with reticular degeneration. H&E: hematoxylin and eosin

**Figure 3:**
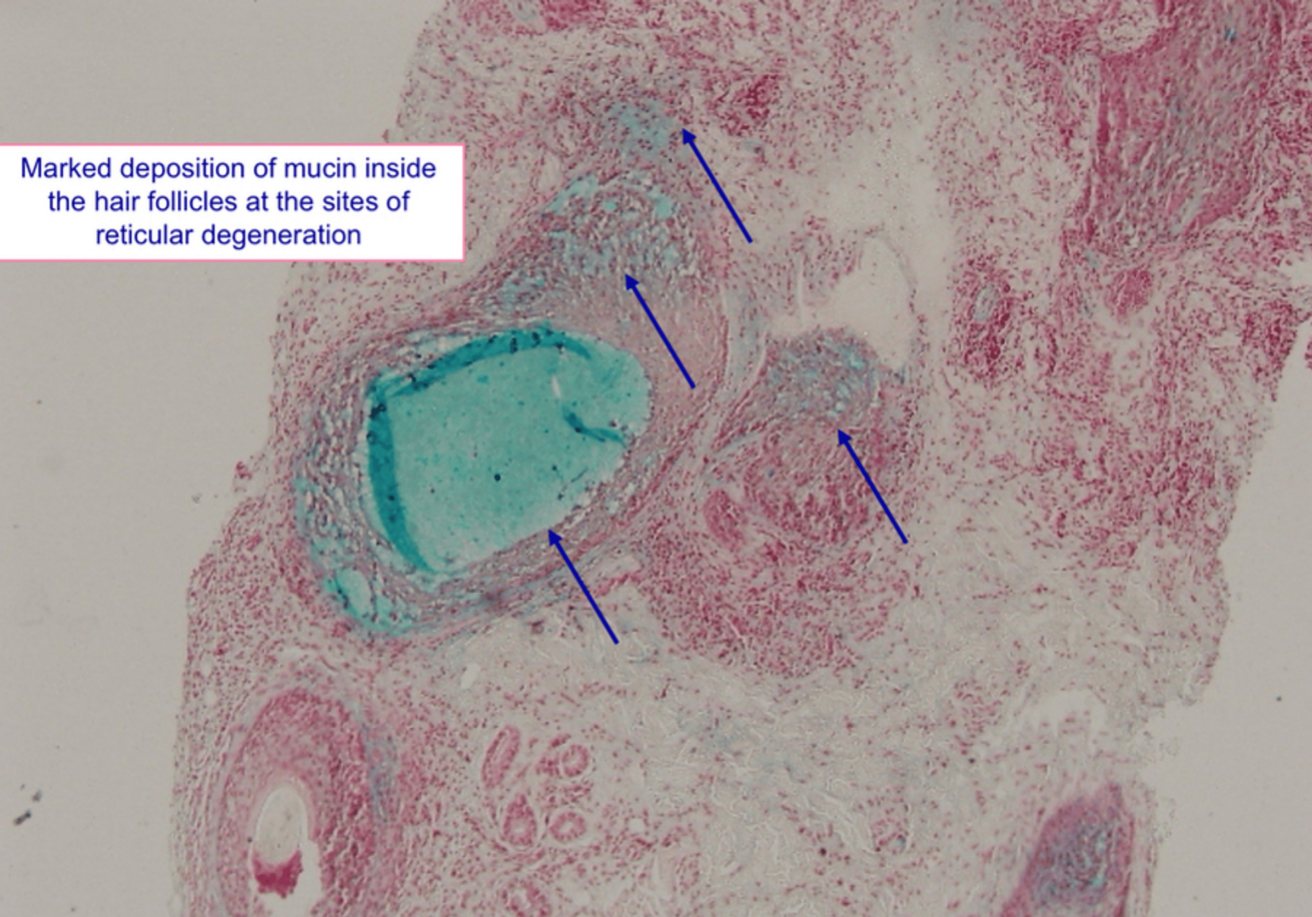
Histopathology (Alcian blue stain) demonstrating mucin deposition in the hair follicle and sebaceous gland (arrows).

A skin biopsy was obtained from the active edge of the lesion for histopathological evaluation. Microscopic examination demonstrated dense perifollicular and perivascular lymphocytic infiltrates extending into the upper and mid-dermis. The follicular epithelium showed intra- and intercellular edema (reticular degeneration) (Figure 2). Special staining with Alcian blue (pH 2.5) confirmed the presence of acid mucopolysaccharide material, consistent with FM (alopecia mucinosa) (Figure 2). There was marked mucin deposition within the outer root sheath and sebaceous glands at the sites of follicular degeneration (Figure 3).

No epidermotropism or atypical lymphoid cells were observed. Immunohistochemical staining revealed a predominance of CD3+ T cells with a normal CD4:CD8 ratio, and no loss of pan-T-cell markers, favoring a benign inflammatory process rather than a cutaneous T-cell lymphoma.

Laboratory investigations, including complete blood count, liver and kidney function tests, and thyroid profile, were all within normal limits. Systemic examination revealed no lymphadenopathy or organomegaly. The overall findings supported the diagnosis of primary (idiopathic) FM.

The patient was started on topical corticosteroids and emollient therapy, with instructions for regular follow-up to monitor for resolution or recurrence. He was also counseled on the importance of long-term observation to exclude potential evolution into a lymphoproliferative process such as folliculotropic MF, although no clinical or histopathologic evidence of malignancy was present at the time of diagnosis.

## Discussion

The pathogenesis of FM remains incompletely understood. Proposed mechanisms include aberrant immune-mediated processes, with circulating immune complexes and cell-mediated immunity implicated in follicular epithelial degeneration. Drug-induced FM has also been described, notably with biologic agents such as adalimumab [4].

FM represents a diagnostic and therapeutic challenge because of its rarity, broad differential diagnosis, and potential malignant associations. In our patient, the clinical presentation of an erythematous, alopecic plaque on the forehead with partial eyebrow involvement persisted for several months, raising an extensive differential diagnosis that included tumid lupus erythematosus, sarcoidosis, cutaneous lymphoma, alopecia areata, fungal infections, and leprosy [4]. Histopathological confirmation of mucin deposition within follicular epithelium and perifollicular lymphocytic infiltrates established the diagnosis.

The distinction between primary and secondary FM is clinically important. Primary FM, often seen in younger individuals, may resolve spontaneously within two months to two years, with minimal morbidity confined to cosmetic concerns. In contrast, secondary FM, particularly when associated with MF or Hodgkin’s disease, carries a significantly higher morbidity and mortality risk [4].

Management strategies are varied, reflecting the unpredictable natural history of the disease. Reported therapies include topical, intralesional, and systemic corticosteroids, photochemotherapy (psoralen plus ultraviolet-A, PUVA), nitrogen mustard, radiation therapy, and immunomodulatory agents such as dapsone, indomethacin, and interferons. However, therapeutic efficacy is difficult to ascertain due to spontaneous remissions and relapses [5,6]. In our patient, the absence of systemic symptoms and histologic atypia favored a diagnosis of primary localized FM, supporting a conservative approach with close dermatologic follow-up.

A critical aspect of management is long-term surveillance. Given the unresolved debate as to whether FM is a precursor lesion to MF or simply a reactive mucinous process, repeating biopsies and vigilant clinical monitoring are essential.

## Conclusions

In conclusion, this case underscores the importance of maintaining a high index of suspicion for FM in young patients presenting with chronic localized alopecic plaques. While primary FM generally carries a favorable prognosis, the potential progression to cutaneous lymphoma necessitates long-term follow-up. Further research into its pathogenesis and natural history is essential to better guide diagnostic and therapeutic strategies.

This case highlights the need to consider FM in the differential diagnosis of chronic, localized alopecic plaques in younger adults. Although primary FM typically follows a benign course, additional biopsy specimens and extremely close follow-ups are crucial in these patients due to the potential progression to cutaneous lymphoma. Ultimately, this case reinforces the need for further research to improve the diagnostic accuracy and guide therapeutic decisions.
